# Clinical implication of Time To Brain Metastasis (TTBM) according to breast cancer subtypes

**DOI:** 10.1186/2193-1801-2-136

**Published:** 2013-03-28

**Authors:** Hee Kyung Ahn, Yeon Hee Park, Su Jin Lee, Silvia Park, Chi Hoon Maeng, Won Park, Doo Ho Choi, Seung Jae Hur, Jin Seok Ahn, Young-Hyuck Im

**Affiliations:** Division of Hematology-Oncology, Department of Medicine Samsung Medical Center, Sungkyunkwan University School of Medicine, 50 Irwon-dong, Gangnam-gu Seoul, 135-710 South Korea; Radiation Oncology, Samsung Medical Center, Sungkyunkwan University School of Medicine, Seoul, South Korea

**Keywords:** Breast cancer, Brain metastases, HER2, Triple negative, Trastuzumab

## Abstract

The aims of the present study were to investigate how breast cancer (BC) subtypes and treatment affect time to brain metastasis (TTBM). We retrospectively investigated 189 consecutive patients who were diagnosed with brain metastasis (BM) from BC between 2000 and 2009 at Samsung Medical Center. We analyzed TTBM from initial diagnosis of metastatic BC according to subtypes and trastzumab (T) administration before BM diagnosis. The median age of 189 BM patients from BC was 48 years. We divided TTBM into four groups considering BC subtypes and treatment; HR-positive/HER2-negative (n=45), HER2-positive with T before BM development (n=47), HER2-positive without T before BM development (n=39), and TNBC (n=58). The median TTBMs for each group were 17.5 months, 13.7 months, 5.8 months, and 2.9 months, respectively (p<0.001). HER2-positive without T (HR 1.892, p=0.008) and TNBC (HR 1.652, p=0.023) were independently associated with shorter TTBM. In multivariate analysis, HER2-positive without T (hazard ratio 1.725, p=0.002) and TNBC (hazard ratio 1.579, p=0.022) were independent risk factors for worse metastatic OS compared with HR-positive/HER2-negative subtype. TTBMs were shorter in patients with HER2-positive without T and TNBC among BC subtypes. Prospective clinical study for high risk patients for early BM is warranted.

**Electronic supplementary material:**

The online version of this article (doi:10.1186/2193-1801-2-136) contains supplementary material, which is available to authorized users.

## Introduction

Breast cancer (BC) is the second-most common cancer that spreads to the brain (Lin et al. 2004). The incidence of brain metastasis (BM) from BC appears to be increasing attributable to improved neuroimaging, increasing numbers of BC patients, and prolonged survival due to improved systemic therapies. Symptomatic BM in patients with metastatic breast cancer (MBC) occurs in 10-16% of patients, revealing up to 30% when autopsy diagnosis of BM is included (Santarelli et al. 2007; Al-Shamy & Sawaya 2009). The median survival after development of BM in BC patients is approximately 4–6 months and 1– and 2-year survival rates are approximately 20% and 2%, respectively.

Traditionally, a number of risk factors for BM from MBC have been reported to be associated with high tumor grade, a negative hormone receptor status, early-onset BC, African-American ethnicity, HER2 overexpression or the presence of lung and liver metastases. The median latency between the initial diagnosis of BC and the onset of BM is 2 to 3 years, suggesting that BM usually occurs late in the course of MBC. It has been shown by Heitz et al. (2009) triple-negative or HER2-positive BC is associated with higher and earlier BM development in the course of disease compared with ER+/HER2- subtype. However, the how BC subtypes predispose to BM differently in their longitudinal disease course and their relation with systemic treatment have not been described well.

Recently it has been reported that the tool as nomogram to predict subsequent BM in patient with MBC with non-BM (Graesslin et al. 2010). And, we have reported that new prognostic model to prediction of outcomes for patients with BM reflecting the different biologic features of BC, including treatment effect and status of extracranial disease control (Ahn et al. 2012) by refining the Sperduto’s BC-specific GPA index (Sperduto et al. 2012) through analysis of a nomogram and through the incorporation of unique biological features of BCs. Thus, we need to incorporate time to BM (TTBM) in addition to selection of enriching patients and prediction prognosis of BM from BC.

We hypothesized that MBC patients may predispose to BM differently during the disease courses according to BC subtypes and treatments, and timing of BM may affect metastatic survival.

In the present study, we aimed to describe how tumor subtype and therapy-related factors of anti-HER2 treatment differently affect TTBM in BC patients. Next, we investigated whether TTBM influence on metastatic overall survival in MBC patients.

## Patients and methods

### Patients’ cohort

**Figure 1:**
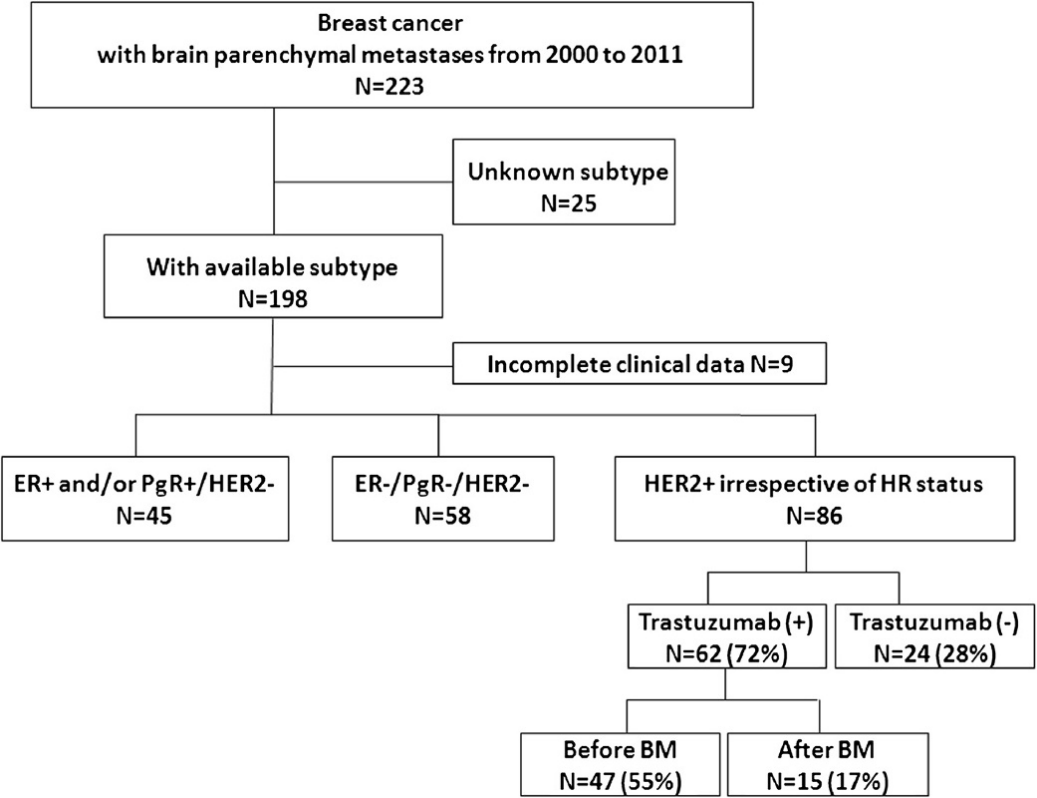
**Patients’ cohort.**

From the data base in our institute, we identified 223 consecutive patients who were diagnosed with BM from BC between 2000 and 2011 at Samsung Medical Center. Among these, we excluded 25 patients who did not have available IHC data of estrogen receptor (ER), progesterone receptor (PgR), or HER2. An additional 9 patients whose clinical data were incomplete were excluded, leaving a final cohort of 189 patients (Figure 1). All patients had histologically confirmed adenocarcinoma of the breast in the primary and/or metastatic sites(s) by two experienced pathologists who determined the primary tumor characteristics. Clinical data including patients characteristics, tumor subtype according to the status of immunohistochemical staining for ER, PgR, and HER2. The status of ER and PgR positivity were defined by Allred scoreof 3–8 by IHC using ER antibody (Immunotech) and PgR antibody (Novocastra), respectively.HER2 status was evaluated using an antibody (DAKO)and/or fluorescence in situ hybridization (FISH).Grades 0 and 1 for HER2 by IHC were defined as negative and grade 3 as positive. In patients with HER2 2+ by IHC, FISH was performed to confirm HER2 amplification. Triple negativity was defined as a lack of ER,PgR, and HER2 expressions.

Routine screening for BM was not performed. BM was diagnosed using brain magnetic resonance imaging (MRI) and/or surgical intervention when clinically suspected. Treatment modalities for BM included symptomatic management with corticosteroids, WBRT, surgical resection, SRS, and/or systemic treatment at physician’s discretion.

### Statistical analysis

Patient characteristics were compared using chi-square and Fisher’s exact test (categorical variables). Time to brain metastases (TTBM) was defined from the date of initial diagnosis of distant metastasis to the date of BM diagnosis. TTBM according to BC subtype and trastuzumab treatment was assessed by Kaplan-Meier methods and compared using log-rank test. Multivariate analysis to assess prognostic factor for TTBM was performed using a Cox-proportional hazards model, and the following factors were included in the model: age at initial diagnosis of primary BC, primary metastatic disease, initial metastatic sites including liver, lung, or bone, BC subtypes. BC subtypes were defined as follows: hormone receptor (HR)-positive type was as ER and/or PgR positive with HER2-negative, HER2-positive type wasHER2-positive irrespective of HR status, TN type was defined as lack of ER, PgR, and HER2 expressions. In addition to this, HER2-positive type was divided into two subtypes according to the treatment of trastuzumab (T): HER2-positive with T and HER2-positive without T. Metastatic overall survival (mOS) was defined from the date of initial diagnosis of distant metastases to the date of death. Metastatic OS was assessed by Kaplan-Meier method and compared using log-rank test according to BC subtypes.

## Results

### Patient characteristics

Patient demographics are displayed in Table 1. Median age at diagnosis of BM was 48 years. Eighty-six percent (n=163) of the patients had invasive ductal carcinoma and the remaining had other pathologies. Ten percent (n=18) of the patients was initially diagnosed as stage IV metastatic disease. Among 189 patients, the proportion of HR-positive/HER2-negaitve, HER2-positive irrespective of HR status, and TNBC was 23.8% (n=45), 46.5% (n=86), and 30.7% (n=58), respectively. Among eighty six HER2-positive patients, thirty seven patients were HR-positive and forty nine patients were HR-negative. Sixty two (72%) of eighty six HER2-positive patients had received anti-HER2 treatment with trastuzumab in their disease course, and the number of patients who received trastuzumab before BM development was forty-seven(55%). Trastuzumab was administered as neoadjuvant treatment in one patient, as adjuvant treatment in twelve patients, and as palliative treatment in remaining patients. Overall survival from initial diagnosis of distant metastases (mOS), and from BM diagnosis (BM-OS) was 23.3 and 9.6 months, respectively.

**Table 1:** **Baseline characteristics of 189 breast cancer patients with brain metastases**

Characteristics	Total N=189 (%)
Median age (range)	
at initial diagnosis of distant metastasis	46 (25–85)
at initial diagnosis of BM	48 (26–87)
Menopausal status at initial diagnosis	
premenopausal	104 (55%)
postmenopausal	42 (22%)
unknown	43 (23%)
Histology	
Invasive ductal carcinoma	163 (86%)
Invasive lobular carcinoma	3 (2%)
Others	10 (5%)
Unknown	13 (7%)
Stage at initial diagnosis	
I/II	81 (43%)
III	76 (40%)
IV	18 (10%)
unknown	14 (7%)
Tumor subtype	
HR-positive/HER2-negative	45 (23%)
HER2-positive irrespective of HR status	86 (47%)
TNBC	58 (31%)
AntiHER2 Treatment among HER2-positive patients (N=86)	
Before BM diagnosis	47 (51%)
as an adjuvant treatment	12
as a neoadjuvant treatment	1
as a palliative treatment	34
After BM diagnosis	15 (17%)
No anti-HER2 treatment	24 (32%)
Overall Survival, median months	
from initial distant metastases (mOS)	23.3
from initial BM diagnosis (BM-OS)	9.6

### Characteristics of BM development according to tumor subtype

We subdivided patients into four groups according to expression of HR and HER2 and trastuzumab administration before BM development. Characteristics according to subtype and trastuzumab treatment are featured in Table 2. Median age at initial BM diagnosis was younger in TNBC (median 45 years) and HER2-positive without trastuzumab (median 46 years) than HR-positive/HER2-negative patients (median 51 years) and HER2-positive with trastuzumab (median 50 years) (p=0.005). Stage at MBC diagnosis, performance status at BM diagnosis, number of brain metastases, site of initial distant metastasis, proportion of concomitant leptomeningeal seeding at initial BM diagnosis were not different among the four subgroups. Brain was an initial metastatic site more frequently in TNBC (38%) or HER2-positive without trastuzumab treatment (37%) compared with in HR-positive/HER2-negative (21%) or HER2-positive with trastuzumab administration before BM (21%). Brain was the only metastatic site in TNBC (21%, n=12) more frequently compared with other subtypes (11%, n=15).

**Table 2:** **Characteristics of brain metastases according to breast cancer subtype and trastuzumab treatment before brain metastases development**

	HR-positive/HER2-negative (N=45)	HER2-positive		TNBC^†^(N=58)	P-value
		Without T^*^(N=39)	With T^*^(N=47)		
Age, median (range)					
At initial distant mets	49 (30–85)	45 (25–68))	46 (33–66)	44 (26–65)	0.145
At BM diagnosis	51 (30–87)	46 (26–70)	50 (34–67)	45 (26–67)	0.005
Stage at initial diagnosis					0.088
I/II	27 (61%)	14 (38%)	15 (30%)	25 (43%)	
III	11 (23%)	19 (48%)	22 (48%)	24 (41%)	
IV	4 (9%)	1 (2%)	7 (15%)	6 (10%)	
unknown	3 (7%)	5 (12%)	3 (7%)	3 (5%)	
Brain as initial metastatic site	9 (21%)	15 (37%)	10 (21%)	22 (38%)	0.112
Brain,the only metastatic site	5 (11%)	4 (10%)	6 (13%)	12 (21%)	0.142
Site of initial distant metastasis					
Bone	18 (47%)	18 (50%)	15 (44%)	21 (38%)	0.705
Lung	14 (37%)	14 (39%)	11 (32%)	26 (47%)	0.535
Liver	5 (13%)	6 (17%)	13 (38%)	8 (15%)	0.036
Pleura	7 (18%)	5 (13%)	5 (16%)	8 (15%)	0.936
LMS^‡^ at BM diagnosis	7 (17%)	6 (15%)	6 (16%)	12 (21%)	0.824
ECOG PS at BM diagnosis					0.482
0-1	32 (72%)	27 (69%)	33 (70%)	35 (60%)	
2	5 (12%)	6 (16%)	10 (22%)	13 (22%)	
3-4	8 (19%)	5 (14%)	4 (9%)	10 (17%)	
Number of brain metastasis					0.430
1	16 (36%)	10 (26%)	8 (17%)	17 (29%)	
2-3	3 (7%)	8(21%)	8 (17%)	9 (16%)	
≥3	24 (53%)	20 (51%)	31 (66%)	31 (53%)	
missing	2 (4%)	1(3%)	0(0%)	1 (2%)	
Extracranial systemic control at BM diagnosis					0.122
PR/SD	7 (16%)	6 (15%)	16 (34%)	9 (16%)	
PD	27 (60%)	25 (64%)	20 (43%)	37 (64%)	
No other systemic mets.	5 (11%)	4 (10%)	6 (13%)	12 (21%)	
unknown	6 (13%)	4 (10%)	5 (11%)	0 (0%)	

^*^T; trastuzumab, ^†^TNBC; triple negative breast cancer, ^‡^LMS; leptomeningeal seeding.

### Time to brain metastases (TTBM)

**Figure 2:**
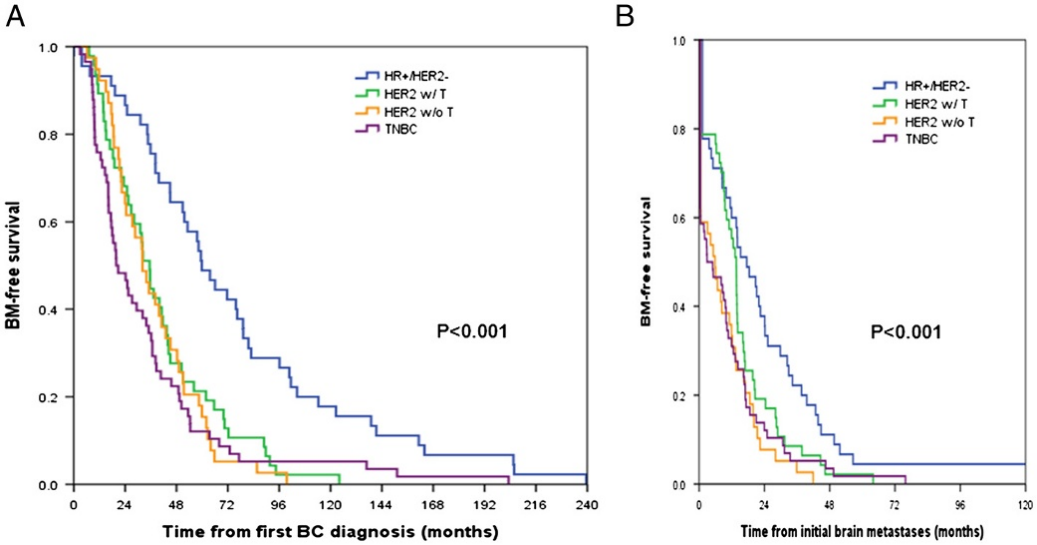
**Time to brain metastases (TTBM) according to breast cancer subtype.** (**A**)Time to brain metastases from diagnosis of primary breast cancer according to subtype and trastuzumab administration before brain metastases development. (**B**)Time to brain metastases from initial diagnosis of metastatic breast cancer according to subtype and trastuzumab administration before brain metastases development.

Times to brain metastases from the primary BC diagnosis or from the metastatic BC were different according to tumor subtype and trastuzumab effect. Median time to brain metastases from initial diagnosis of primary BC was 19.9 months in TNBC, 32.1 months in HER2-positive without trastuzumab, 35.4 months in HER2-positive with trastuzumab, and 59.8 months in HR-positive/HER2-negative patients, respectively (p<0.001, Figure 2A). Median time to brain metastases from diagnosis of distant metastases was 2.9 months in TNBC, 5.8 months in HER2-positive without trastuzumab, 13.7 months in HER2-positive with trastuzumab, 17.5 months with HR-positive/HER2-negative, respectively (p=0.001, Figure 2B).

In multivariate analysis, HER2-positive without trastuzumab (hazard ratio 1.892, p=0.008) and TNBC (hazard ratio 1.652, p=0.023) were independent prognostic factors for shorter TTBM compared with HR-positive/HER2-negative subtype. Patients presenting with bone metastases as first distant metastatic sites was associated with longer TTBM (hazard ratio 0.695, p=0.033), significantly. Age and primary metastatic disease, were not associated with TTBM in multivariate analysis (Table 3).

**Table 3:** **Cox-regression multivariate analysis of predictive factors for time to brain metastases (TTBM) from initial distant metastases**

Characteristics	HR (95% C.I.)	P-value
**Age at first diagnosis of primary disease**	1.001 (0.985-1.018)	0.863
**Stage IV at initial diagnosis of primary BC**	0.843 (0.475-1.495)	0.559
**Site of initial distant metastases**		
Bone	0.695 (0.498-0.969)	0.033
Lung	1.138 (0.811-1.597)	0.453
Liver	1.431 (0.934-2.190)	0.099
**Subtype with trastuzumab effect**		
HR-positive/HER2-negative	reference	
HER2-positive withoutTrastuzumab	1.892 (1.177-3.040)	0.008
HER2-positive with Trastuzumab	1.064 (0.650-1.741)	0.656
TNBC	1.652 (1.071-2.546)	0.023

### Metastatic overall survival according to BC subtypes and trastuzuamb effect

**Figure 3:**
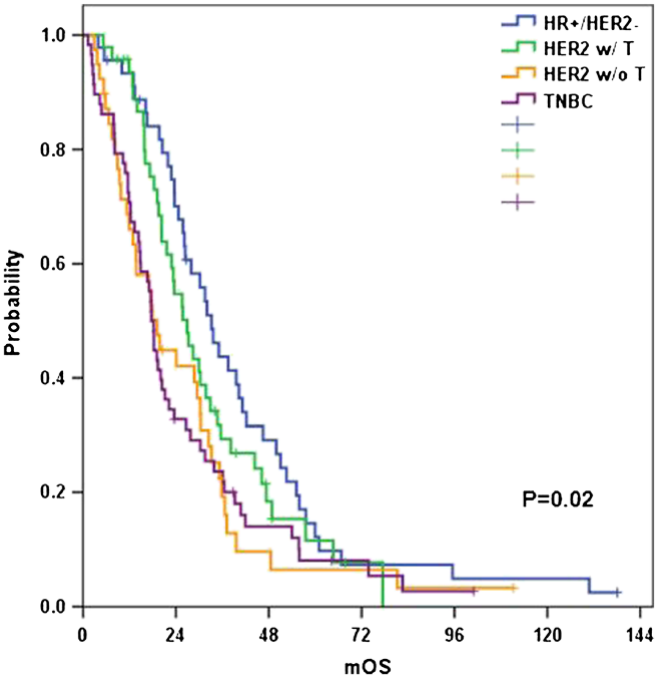
**Overall survival from the initial diagnosis of distant metastases according to breast cancer subtype.**

Metastatic overall survivals from the date of diagnosis of distant metastasis to death were significantly different according to the four BC subtypes incorporated with trastuzumab effect (Figure 3). Median time to death from distant metastases (mOS) was 17.6 months in TNBC, 19.1 months in HER2-positive without trastuzumab, 26.9 months in HER2-positive with trastuzumab, and 33.2 months in HR-positive/HER2-negative patients, respectively (p=0.020, Figure 3). In multivariate analysis, HER2-positive without trastuzumab (hazard ratio 1.725, p=0.002) and TNBC (hazard ratio 1.579, p=0.022) were independent risk factors for worse metastatic OS compared with HR-positive/HER2-negative subtype.

## Discussion

Recent improvements of systemic treatments including new cytotoxic agents and third generation aromatase inhibitors (AI) have brought to longer survival in patients with MBC. Prominently, incorporation of targeted therapies, such as trastuzumab, in HER2-positive BC has changed the natural history of this disease, prolonging survival. Consequently, many women now survive long enough to develop CNS disease. It also has emphasized local treatment modalities, such as whole brain radiation, stereotactic surgery, and tumor removal (Chargari et al. 2010; Suh 2010; Kased et al. 2009; Muacevic et al. 2004).

With BM occurring as the result of poor systemic disease control, predicting the timing of BM development may provide rationale for early intervention and treating BM considering prognostic stratification of BM. This retrospective single-center study analyzing TTBM according to BC subtype and anti-HER2 treatment trastuzumab was based on a relatively large cohort of patients with BM from BC. The distributions of HER2-positive and TNBC among BM patients from BC were higher than in those of the general proportion of BC patients, which reflects predilection to BM of HER2-positive and TNBC BCs (Slimane et al. 2004; Sanna et al. 2007; Ryberg et al. 2005; Lin et al. 2008; Heitz et al. 2009; Pestalozzi et al. 2006). In terms of TTBM from initial diagnosis of metastatic disease, median TTBM was significantly shorter in TNBC patients (2.9 months) and HER2-positive without trastruzumab treatment (5.8 months) compared patients with HR-positive/HER2-negative (17.5 months) or HER2-positive patients who received trastuzumab (13.7 months). This propensity of HER2-positive and TNBC to early BM occurrence is consistent with previous studies by Heitz et al. (2009).This result is also supported by other translational results (Sarrio et al. 2008; Lin et al. 2004; Duchnowska et al. 2009). However, they did not investigate anti-HER2 treatment effect on TTBM. In the present study, TTBM from initial primary BC was not different according to trastuzumab treatment but TTBM from initial diagnosis of distant metastases was significantly longer in HER2-positive patients with trastuzumab treatment. In multivariate analysis, both HER2-positive without trastuzumab treatment and TNBC were independently associated with earlier BM occurrence.

Prognostic model for BM from BC patients were reported and there is biologic evidence for higher propensity for BM in HER2-positive and TNBC patients (Ahn et al. 2012; Heitz et al. 2009; Sperduto et al. 2012), although routine screening of BM for high risk patients did not show definite survival benefit (Niwinska et al. 2007).While general therapeutic nihilism should be avoided, it is still important to recognize that the number of BM, the extent of the systemic disease, and also the BC subtype have to be taken into account when choosing individual treatment regimens. Finally, special emphasis will be put on established and future approaches to prevent BM. Incorporation of TTBM to prediction of prognosis in BM from BC patients may facilitate screening at most risky period, potential development of possible prophylactic strategies, and choice of treatment modalities, which are ready for prospective clinical trial.

This study is limited in that the study population is not the entire patients with BC diagnosis, but the patients who diagnosed with BM from BC, and therefore we could not evaluate the actual incidence of BM exactly. Instead, we have evaluated the TTBM from the time of first diagnosis of metastatic BC according to subtype and targeted treatment among BM patients.

Considering high cost of screening brain MRI, the prospective clinical trial selecting the patients’ population at high risk of BM who has significant benefit from screening and treating asymptomatic BM is crucial. Given that over 50% of BM in HER2-positive and TNBC occur in the first year after diagnosis of metastatic BC in this study which is compatible with previous reports (Heitz et al. 2009), it might be reasonable to confine candidates to screen asymptomatic BM to HER2-positive and TNBC population, for more risky period after initial diagnosis of metastatic disease, and screening should be considered incorporated with systemic disease control. Moreover, in these cases of BM development with well controlled extracranial systemic disease, the optimal treatment strategy is questionable whether systemic chemotherapy regimen should be changed or maintained and how local therapy should be combined with systemic treatments, especially for patients with BM as the only progression site.

## Conclusions

The present study identified HER2-positiveBC without trastuzumab treatment and TNBC as independent risk factors for shorter TTBM from the initial distant metastasis. Incorporating prognostic index, TTBM may provide the rational approach to plan prospective clinical trial whether there is a population in which screening at most risky period or potential prophylactic strategies for BM could have clinical benefit.

## Authors’ original submitted files for images

Below are the links to the authors’ original submitted files for images. Authors’ original file for figure 1 Authors’ original file for figure 2 Authors’ original file for figure 3

## References

[CR1] Ahn HK, Lee S, Park YH, Sohn JH, Jo JC, Ahn JH, Jung KH, Park S, Cho EY, Lee JI, Park W, Choi DH, Huh SJ, Ahn JS, Kim SB, Im YH (2012). Prediction of outcomes for patients with brain parenchymal metastases from breast cancer (BC): a new BC-specific prognostic model and a nomogram. Neuro Oncol.

[CR2] Al-Shamy G, Sawaya R (2009). Management of brain metastases: the indispensable role of surgery. J Neurooncol.

[CR3] Chargari C, Campana F, Pierga JY, Vedrine L, Ricard D, Le Moulec S, Fourquet A, Kirova YM (2010). Whole-brain radiation therapy in breast cancer patients with brain metastases. Nat Rev Clin Oncol.

[CR4] Duchnowska R, Dziadziuszko R, Czartoryska-Arlukowicz B, Radecka B, Szostakiewicz B, Sosinska-Mielcarek K, Karpinska A, Staroslawska E, Kubiatowski T, Szczylik C (2009). Risk factors for brain relapse in HER2-positive metastatic breast cancer patients. Breast Cancer Res Treat.

[CR5] Graesslin O, Abdulkarim BS, Coutant C, Huguet F, Gabos Z, Hsu L, Marpeau O, Uzan S, Pusztai L, Strom EA, Hortobagyi GN, Rouzier R, Ibrahim NK (2010). Nomogram to predict subsequent brain metastasis in patients with metastatic breast cancer. J Clin Oncol.

[CR6] Heitz F, Harter P, Lueck HJ, Fissler-Eckhoff A, Lorenz-Salehi F, Scheil-Bertram S, Traut A, du Bois A (2009). Triple-negative and HER2-overexpressing breast cancers exhibit an elevated risk and an earlier occurrence of cerebral metastases. Eur J Cancer.

[CR7] Kased N, Binder DK, McDermott MW, Nakamura JL, Huang K, Berger MS, Wara WM, Sneed PK (2009). Gamma Knife radiosurgery for brain metastases from primary breast cancer. Int J Radiat Oncol Biol Phys.

[CR8] Lin NU, Bellon JR, Winer EP (2004). CNS metastases in breast cancer. J Clin Oncol.

[CR9] Lin NU, Claus E, Sohl J, Razzak AR, Arnaout A, Winer EP (2008). Sites of distant recurrence and clinical outcomes in patients with metastatic triple-negative breast cancer: high incidence of central nervous system metastases. Cancer.

[CR10] Muacevic A, Kreth FW, Tonn JC, Wowra B (2004). Stereotactic radiosurgery for multiple brain metastases from breast carcinoma. Cancer.

[CR11] Niwinska A, Tacikowska M, Pienkowski T (2007). Occult brain metastases in HER2-positive breast cancer patients: frequency and response to radiotherapy. Acta Oncol.

[CR12] Pestalozzi BC, Zahrieh D, Price KN, Holmberg SB, Lindtner J, Collins J, Crivellari D, Fey MF, Murray E, Pagani O, Simoncini E, Castiglione-Gertsch M, Gelber RD, Coates AS, Goldhirsch A (2006). Identifying breast cancer patients at risk for Central Nervous System (CNS) metastases in trials of the International Breast Cancer Study Group (IBCSG). Ann Oncol.

[CR13] Ryberg M, Nielsen D, Osterlind K, Andersen PK, Skovsgaard T, Dombernowsky P (2005). Predictors of central nervous system metastasis in patients with metastatic breast cancer. A competing risk analysis of 579 patients treated with epirubicin-based chemotherapy. Breast Cancer Res Treat.

[CR14] Sanna G, Franceschelli L, Rotmensz N, Botteri E, Adamoli L, Marenghi C, Munzone E, Cossu Rocca M, Verri E, Minchella I, Medici M, Catania C, Magni E, Goldhirsch A, Nole F (2007). Brain metastases in patients with advanced breast cancer. Anticancer Res.

[CR15] Santarelli JG, Sarkissian V, Hou LC, Veeravagu A, Tse V (2007). Molecular events of brain metastasis. Neurosurg Focus.

[CR16] Sarrio D, Rodriguez-Pinilla SM, Hardisson D, Cano A, Moreno-Bueno G, Palacios J (2008). Epithelial-mesenchymal transition in breast cancer relates to the basal-like phenotype. Cancer Res.

[CR17] Slimane K, Andre F, Delaloge S, Dunant A, Perez A, Grenier J, Massard C, Spielmann M (2004). Risk factors for brain relapse in patients with metastatic breast cancer. Ann Oncol.

[CR18] Sperduto PW, Kased N, Roberge D, Xu Z, Shanley R, Luo X, Sneed PK, Chao ST, Weil RJ, Suh J, Bhatt A, Jensen AW, Brown PD, Shih HA, Kirkpatrick J, Gaspar LE, Fiveash JB, Chiang V, Knisely JP, Sperduto CM, Lin N, Mehta M (2012). Effect of tumor subtype on survival and the graded prognostic assessment for patients with breast cancer and brain metastases. Int J Radiat Oncol Biol Phys.

[CR19] Suh JH (2010). Stereotactic radiosurgery for the management of brain metastases. N Engl J Med.

