# Supplementary figures and images for: Clinical implication of Time To Brain Metastasis (TTBM) according to breast cancer subtypes
Source: Springerplus. 2013 Mar 28;2(1):136. doi: 10.1186/2193-1801-2-136 (PMC3647103; doi:10.1186/2193-1801-2-136)

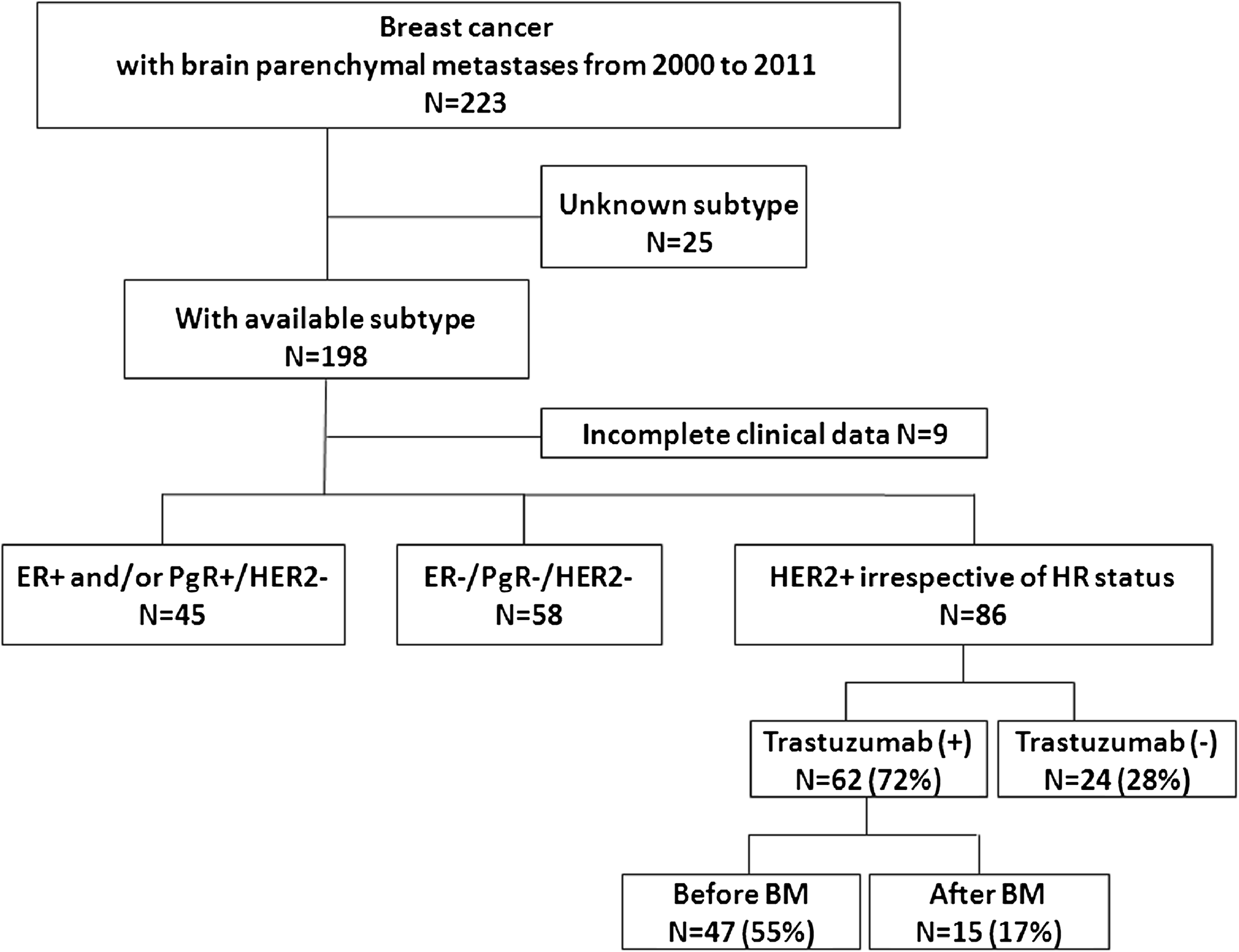

Supplement: Supplementary file 1 — Authors’ original file for figure 1 [file 40064_2013_225_MOESM1_ESM.tiff]

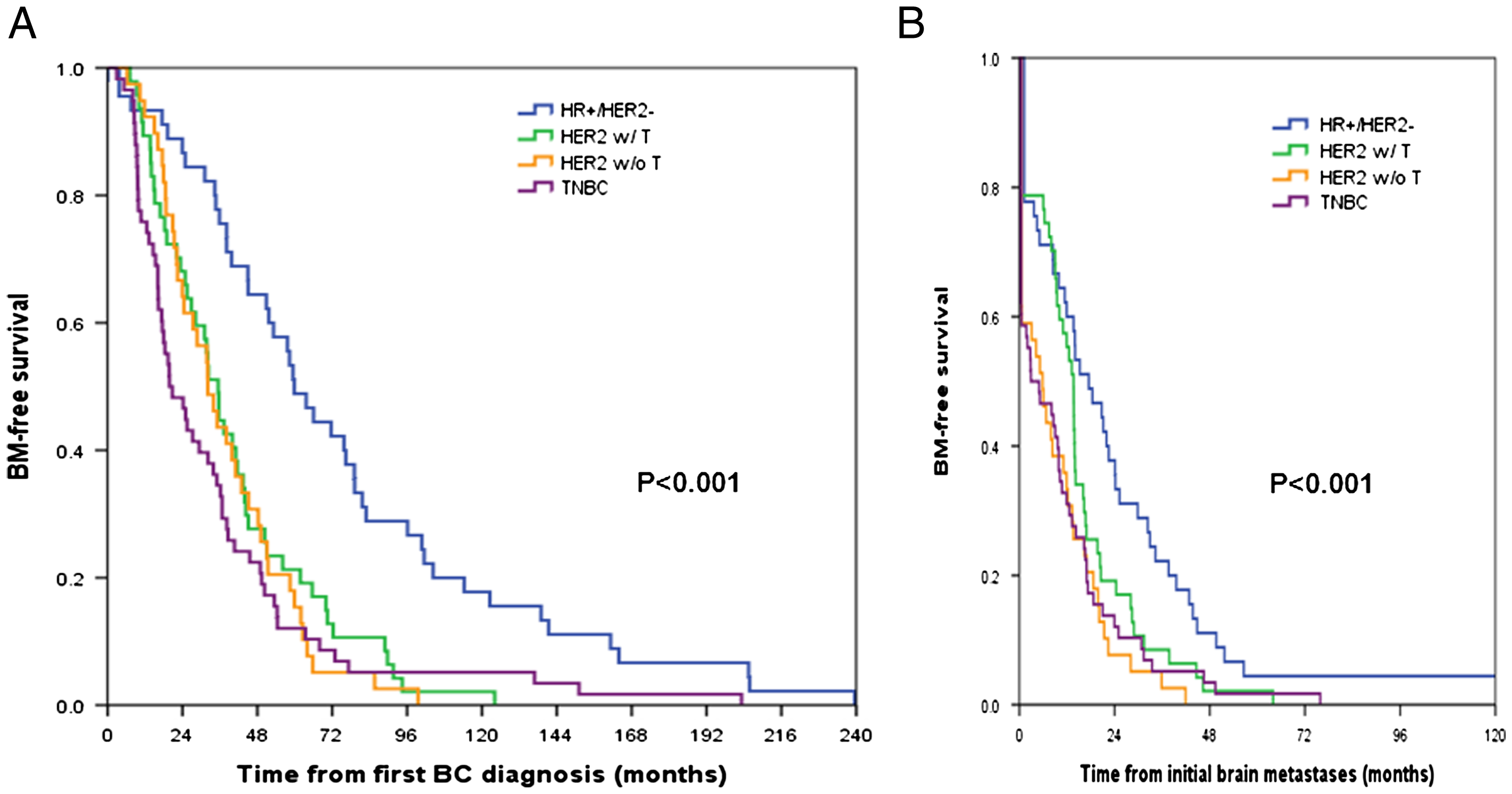

Supplement: Supplementary file 2 — Authors’ original file for figure 2 [file 40064_2013_225_MOESM2_ESM.tiff]

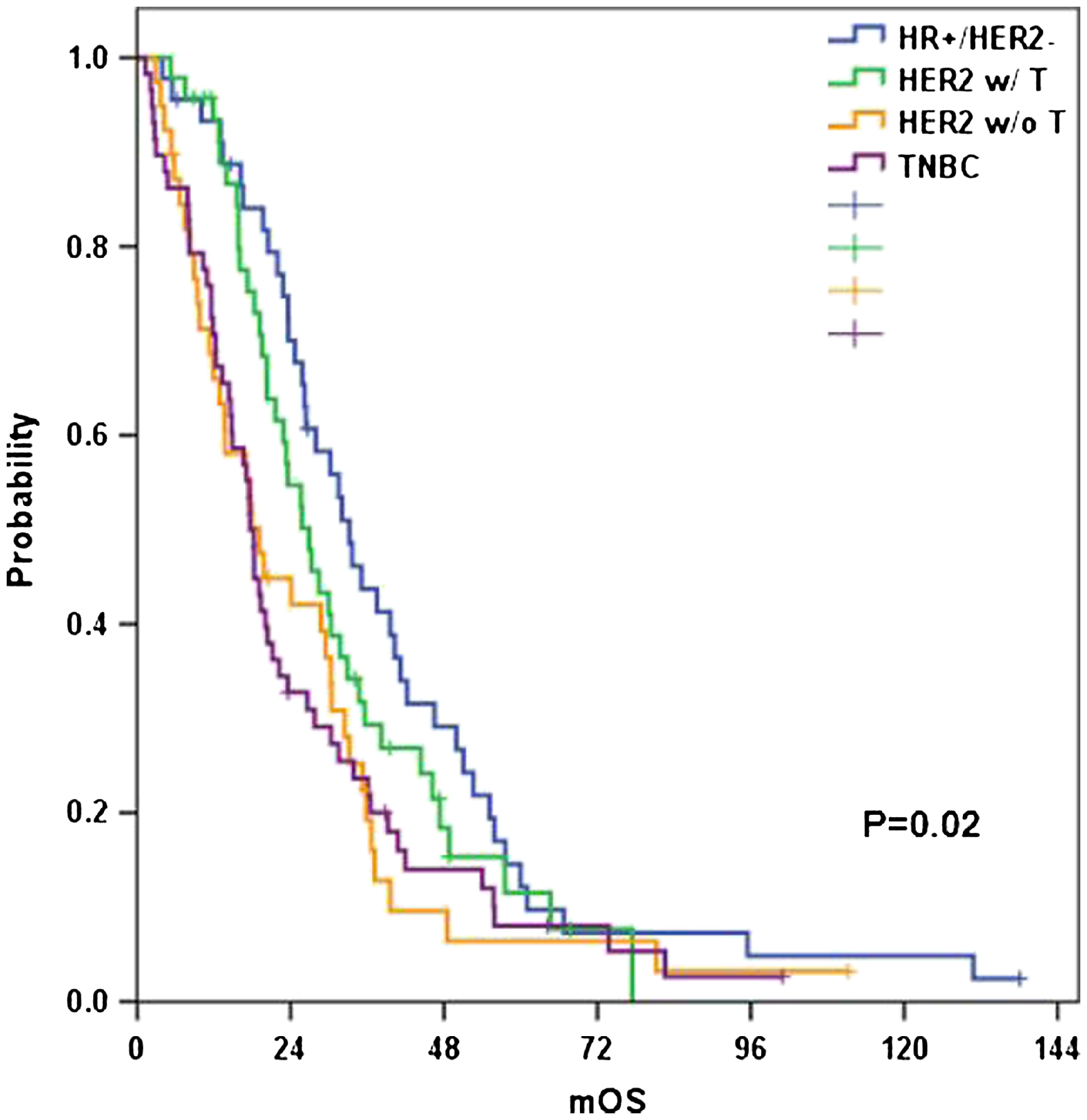

Supplement: Supplementary file 3 — Authors’ original file for figure 3 [file 40064_2013_225_MOESM3_ESM.tiff]
